# Genotype-phenotype correlation and founder effect analysis in southeast Chinese patients with sialidosis type I

**DOI:** 10.1186/s13023-024-03378-5

**Published:** 2024-09-30

**Authors:** Yi-Chu Du, Ling-Han Ma, Quan-Fu Li, Yin Ma, Yi Dong, Zhi-Ying Wu

**Affiliations:** 1 Department of Medical Genetics and Center for Rare Diseases, Second Affiliated Hospital, Zhejiang University School of Medicine and Zhejiang Key Laboratory of Rare Diseases for Precision Medicine and Clinical Translation, Hangzhou, Zhejiang 310009 China; 2 State Key Laboratory of Transvascular Implantation Devices, Hangzhou, Zhejiang 310009 China; 3 Nanhu Brain-computer Interface Institute, Hangzhou, Zhejiang China; 4 MOE Frontier Science Center for Brain Science and Brain-machine Integration, School of Brain Science and Brain Medicine, Zhejiang University, Hangzhou, Zhejiang China

**Keywords:** Sialidosis type I, *NEU1*, Founder effect, Haplotype analysis, Chinese

## Abstract

**Background:**

Sialidosis type 1 (ST-1) is a rare autosomal recessive disorder caused by mutation in the *NEU1* gene. However, limited reports on ST-1 patients in the Chinese mainland are available.

**Methods:**

This study reported the genetic and clinical characteristics of 10 ST-1 patients from southeastern China. A haplotype analysis was performed using 21 single nucleotide polymorphism (SNP) markers of 500 kb flanking the recurrent c.544 A > G in 8 families harboring the mutation. Furthermore, this study summarized and compared previously reported ST-1 patients from Taiwan and mainland China.

**Results:**

Five mutations within *NEU1* were found, including two novel ones c.557 A > G and c.799 C > T. The c.544 A > G mutation was most frequent and identified in 9 patients, 6 patients were homozygous for c.544 A > G. Haplotype analysis revealed a shared haplotype surrounding c.544 A > G was identified, suggesting a founder effect presenting in southeast Chinese population. Through detailed assessment, 52 ST-1 patients from 45 families from Taiwan and mainland China were included. Homozygous c.544 A > G was the most common genotype and found in 42.2% of the families, followed by the c.544 A > G/c.239 C > T compound genotype, which was observed in 22.2% of the families. ST-1 patients with the homozygous c.544 A > G mutation developed the disease at a later age and had a lower incidence of cherry-red spots significantly.

**Conclusion:**

The results contribute to gaps in the clinical and genetic features of ST-1 patients in southeastern mainland China and provide a deeper understanding of this disease to reduce misdiagnosis.

## Introduction

Sialidosis is an autosomal recessive disorder caused by pathogenic mutations in the *NEU1* gene, which is localized on chromosome 6p21.3 and encodes neuraminidase 1 (NEU1). In 1977, Cantz et al. and Spranger et al. recognized the disorder as a syndrome characterized by ataxia, myoclonus, seizures and hypotrophy [1, 2]. According to the clinical manifestation and onset age, sialidosis can be classified into two types [3]. Sialidosis type I (ST-1) usually has an adolescent-onset or adult-onset and presents movement disorders such as progressive myoclonus epilepsy (PME), vision impairment and macular cherry red spots. Epilepsy seizures with polyspike have occasionally been reported in ST-1 patients. ST-1 patients usually exhibit no dysmorphia. Sialidosis type II (ST-2) has an earlier onset and characterized by developmental dysmorphia, hepatosplenomegaly, mental retardation, dysostosis multiplex and macular cherry-red spots. Mutations occurring at different residuals of neuraminidase might explain why the disease presents different phenotypes [4]. Variants of ST-1 patients are considered to result in neuraminidases retaining residual activity, while variants of ST-2 patients would lead to relatively more inactive enzymes [5]. Sialidosis is a rare disorder, with a recent study in Australia reporting a prevalence of 1.4 cases per million individuals [6]. ST-1 is even rarer than ST-2, with fewer than 100 patients reported. Given its rarity and clinical heterogeneity, it is vital to expand the understanding of this disease to minimize misdiagnoses.

More than 40 mutations in *NEU1* have been reported to cause ST-1 worldwide [7–9]. According to the previous research, the two most common genotypes were homozygous c.544 A > G and compound heterozygous c.544 A > G and c.239 C > T, comprising 23.4% and 10.4%, respectively, worldwide; all the ST-1 patients with these two mutations were from Taiwan and mainland China [7–9]. However, most of the mutations were quite rare and private. In addition to the two mentioned genotypes, each of the other genotypes could only account for approximately 1.3-3.9% of the ST-1 patients in different populations, such as in European and American ST-1 populations [9]. Overall, the mutation c.544 A > G was the most frequently reported mutation and exhibited a restricted distribution within the Chinese population. It can appear as frequently as 100% in Taiwan and accounts for 75% of ST-1 patients in mainland China. No distinct mutations in *NEU1* in European or American ST-1 patients were as prevalent as c.544 A > G in Chinese.

Taking together, these findings suggest that the c.544 A > G might have a founder effect in the Chinese population. Accordingly, in this study, we conducted a haplotype analysis of the c.544 A > G to investigate the potential founder effect for the Chinese ST-1 patients. In the past 8 years, we identified 10 ST-1 families. We aimed to provide a valuable reference for the genetic and clinical analysis of sialidosis in China and worldwide. In addition, to the best of our knowledge, the current study comprises the largest group of ST-1 in mainland China.

## Methods

### Study design and participants

Ten ST-1 patients were identified between April 2015 and June 2023. Patients were enrolled from the Second Affiliated Hospital of Zhejiang University, and the study was approved by the ethics committee of the hospital. Each participant or the guardian signed an informed consent form. Patients were diagnosed with ST-1 according to clinical characteristics and genetically confirmed by genetic testing of the *NEU1* gene. The demographic and clinical data were collected, including blood examinations, electroencephalogram (EEG), electromyography (EMG) and magnetic resonance imaging (MRI). In addition, one patient (Patient 6) also underwent 11 C-2βcarbomethoxy-3β-(4-fluorophenyl) tropane (CFT) positron-emission tomography (PET) to measure striatal dopamine transporter (DAT) availability.

## Genetic analysis

Using standard procedures, genomic DNA was extracted from peripheral blood by a QIAamp DNA Blood Minikit (QIAGEN, Hilden, Germany) as previously reported [10]. The mutations in *NEU1* were screened by whole-exome Illumina sequencing (WES) and subsequently confirmed by Sanger sequencing, and the protocol was described in detail in our previous study [11]. Single nucleotide polymorphisms (SNPs) of 500 kb flanking the variant c.544 A > G (6_31325436–32330683) were obtained from the data of Southern Han Chinese (CHS) population based on the 1000 Genomes and human assembly GRCh37. Haploview 4.2 software was used to perform LD analysis. Based on *r*^2^ > 0.8 and minor allele frequency (MAF) > 0.3, 22 tag SNPs were ultimately selected, including 10 sites upstream of c.544 A > G and 12 downstream. Twenty SNPs were genotyped using the Agena Massarray Platform and two SNPs (rs204883 and rs812561) were detected through the Kompetitive Allele-Specific PCR (KASP) typing method using the AB Real-Time PCR System. Sanger sequencing was used as a supplemental sequencing method. However, rs812561 could not be genotyped and ultimately 21 SNPs were included in the analysis (Table 1). In patients who were compound heterozygous for c.544 A > G, their parents were also genotyped to reconstruct the haplotypes. As one patient was out of stock of the DNA sample, eventually 8 patients and six pairs of parents ultimately underwent the haplotype genotyping.

**Table 1:**
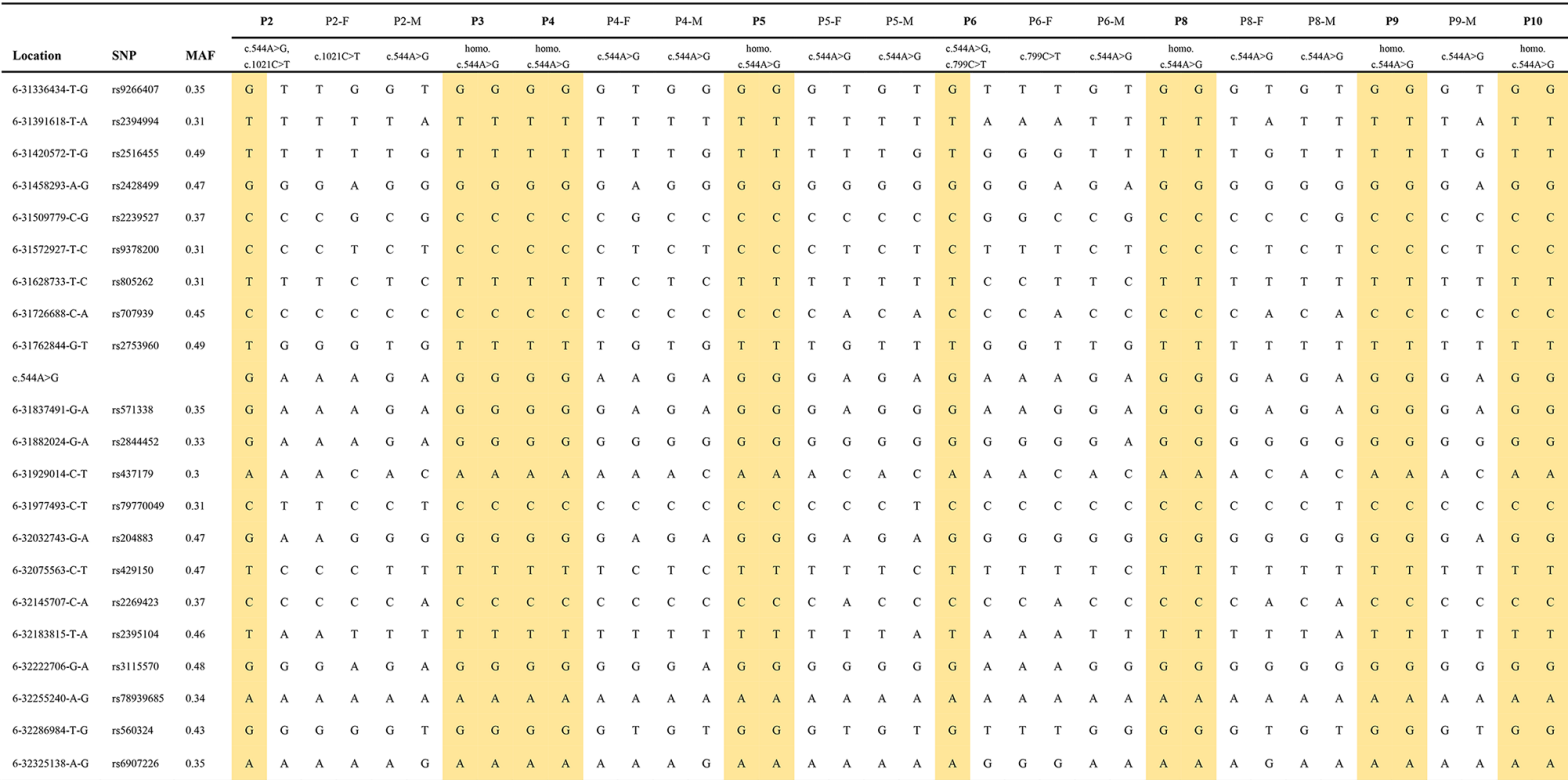
Haplotype analysis in ST-1 patients with c.544 A > G

## Literature review and statistical analysis

To summarize the genotype-phenotype correlation of ST-1 patients in Taiwan and mainland China, articles of sialidosis were reviewed in PubMed (http://www.ncbi.nlm.nih.gov/pubmed/) up to October 2023, and “sialidosis” was used as a keyword in the title or abstract. A detailed review was subsequently conducted to screen ST-1 reports from Taiwan and mainland China. ST-1 patients with confirmed onset age, ethnicity and genetic mutations in the *NEU1* gene were included, and duplicate patients from different analyses were excluded as much as possible. Since sialidosis was rare, if ST-1 patients were of the same sex, onset age or genetic mutation and were from the same medical center, they were considered as duplicate cases. SPSS 23.0 and Microsoft Excel 2016 were used to analyze the data.

## Results

### Genetic features of ten ST-1 patients

**Fig. 1:**
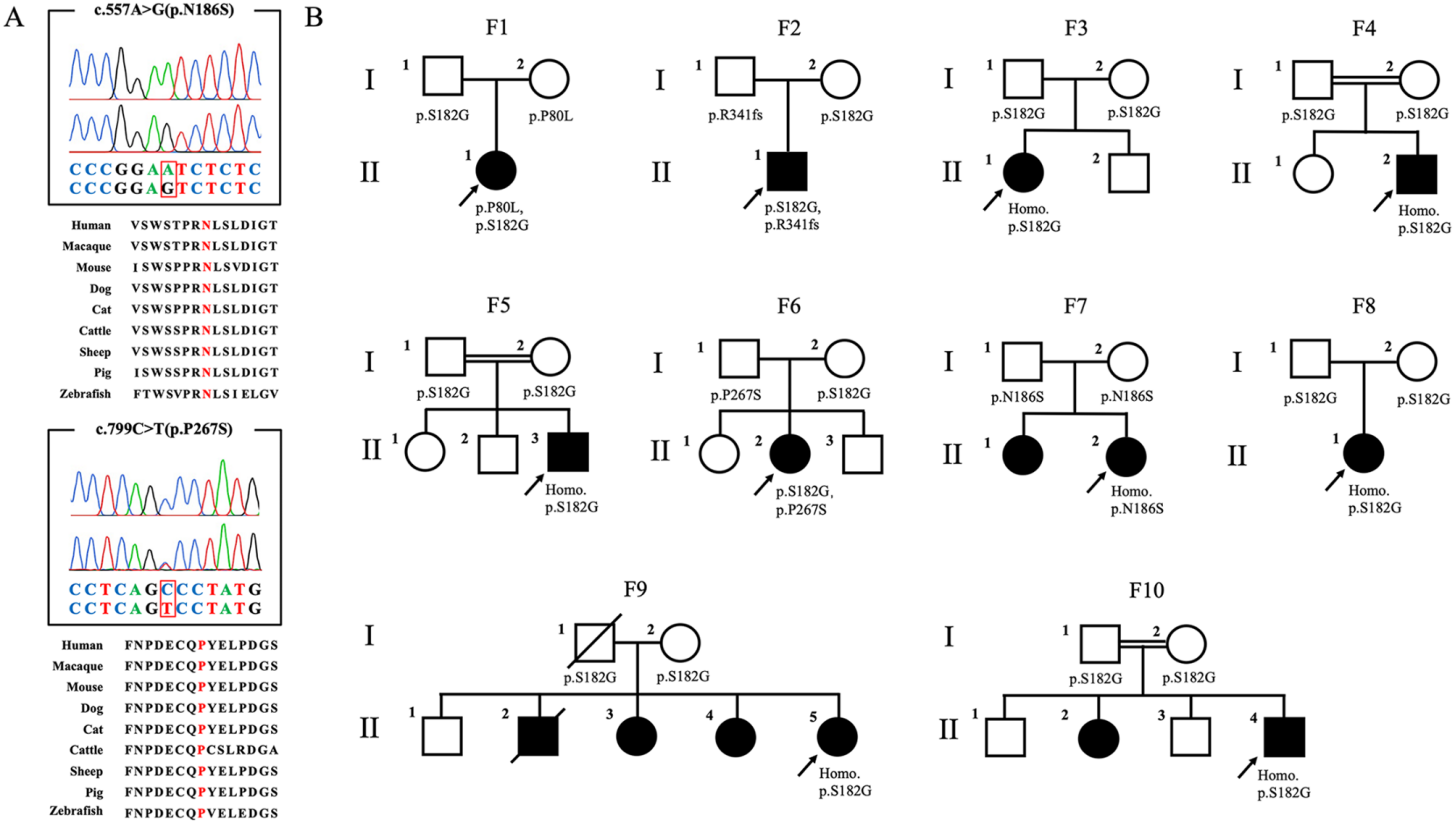
Genetic analysis and pedigree chart. (**A**) Sequencing chromatograms and homology comparison of two novel variants in *NEU1*. The upper chromatogram represents the reference sequence, and the lower chromatogram represents the mutant sequence. Protein sequence alignments of *NEU1* homologues were obtained for two novel variants. The mutated residues are labelled in red. All residues are highly conserved among the different species. (**B**) Pedigree chart of ten ST-1 families. The arrows indicate the proband, and the diagonal lines indicate deceased members. Squares indicate males, and circles indicate females. The black squares or circles indicate affected individuals. Two horizontal lines indicated a consanguineous union

Five mutations were found in 10 probands, including two novel ones (c.557 A > G and c.799 C > T) highly conserved among the different species (Fig. 1A) and three reported ones (c.544 A > G, c.239 C > T and c.1021 C > T). Nine probands harbored the c.544 A > G mutation and six had homozygous c.544 A > G. The variants c.239 C > T, c.1021 C > T and c.799 C > T were found in three other patients with heterozygous c.544 A > G. The remaining patient had the novel homozygous c.557 A > G. The co-segregation results for mutations from the 10 families were shown in Fig. 1B.

The genetic analysis revealed that c.544 A > G was the most common mutation in our cohort. Due to the high frequency (75%) of this mutation, haplotype analysis was performed in 8 families with the c.544 A > G mutation. Twenty-one tSNPs were genotyped. Finally, as shown in Tables 1 and 14 alleles containing the c.544 A > G mutation shared one haplotype, suggesting a founder effect of the c.544 A > G mutation in *NEU1* presenting in southeast Chinese population.

### Clinical characteristics of ten ST-1 patients

A detailed description of the ten unrelated patients was depicted in Table 2. Among them, three were males and seven were females. Six probands were from Zhejiang province, two from Fujian province, one from Guangdong and one from Jiangsu. Therefore, all the ten families were from southeastern China. None of the patients had a remarkable history. As shown in Fig. 1B, three probands had consanguineous parents.

**Fig. 2:**
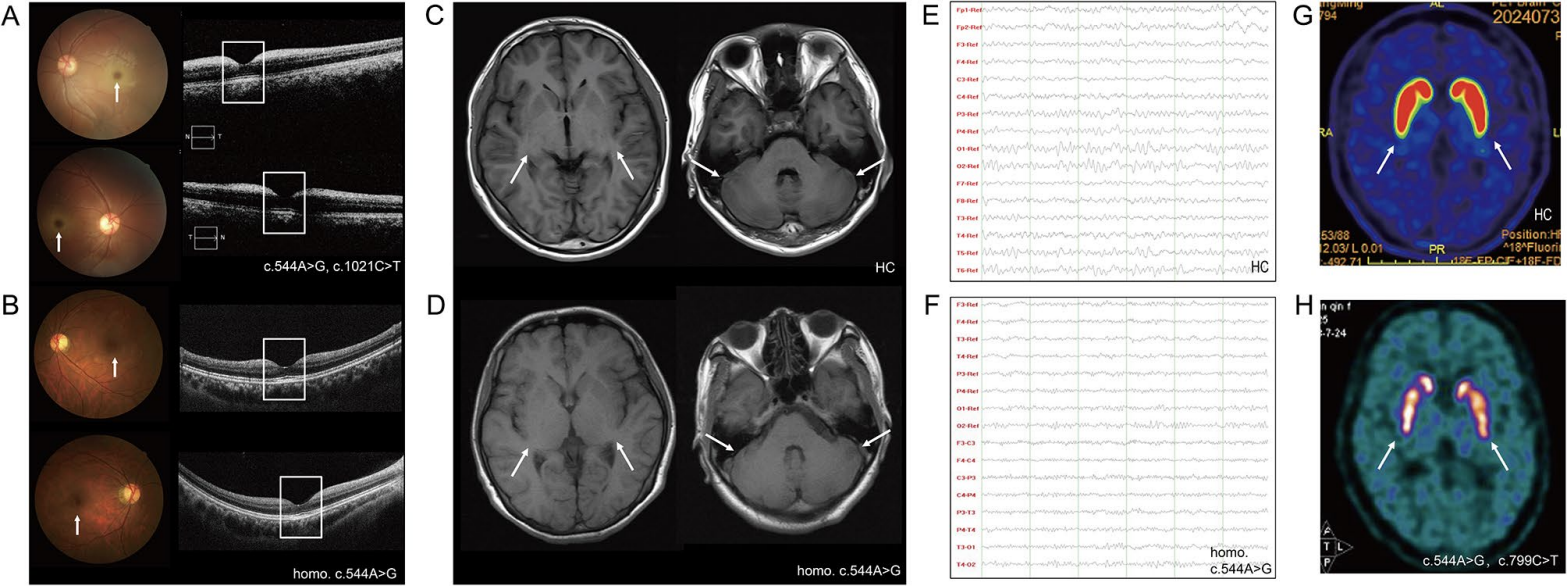
Examination results. (**A**) Fundus examination showed distinct bilateral cherry-red spots in Patient 2, who had the c.544 A > G, c.1021 C > T genotype. The white arrow indicated the CRS. Optical coherence tomography revealed bilateral diffuse weakness of the retinal nerve fiber layer and ganglion cell complex, and increased reflectivity of the macular inner layer (white rectangular box). (**B**) No typical cherry-red spots were observed in Patient 8 with the homozygous c.544 A > G genotype. (**C**) The axial T1 brain magnetic resonance imaging (MRI) of the healthy control was normal. (**D**) The axial T1 brain MRI of the patient with the homozygous c.544 A > G genotype was normal. The white arrow indicated basal ganglion and cerebellum. (**E**) The electroencephalogram (EEG) of the healthy control was normal. (**F**) The EEG of the patient with the homozygous c.544 A > G genotype showed no obvious abnormalities. (**G**) The DAT-FDG PET result of the healthy control was normal. (**H**) The DAT-FDG PET result suggested that the distributions of dopamine transporters in the bilateral caudate nucleus and putamen were not obviously abnormal in Patient 6. The white arrow indicated the basal ganglion. *HC* healthy control

The average age at onset (AAO) was 20.8 ± 8.4 years (range, 7–36) among the ten patients. The mean age at diagnosis (AAD) was 34.9 ± 12.6 years (range, 13–53). The average diagnosis time was 14.9 years. Gait ataxia was the most common initial symptom, found in 5 patients, followed by myoclonus complained by four patients, vision impairment by two and nystagmus by one as initial symptom. During the disease duration, 6 patients experienced myoclonus, and one had generalized tonic-clonic seizures. Vision impairment was reported by four patients during illness and two out of 7 patients who underwent ophthalmic examination exhibited macular cherry-red spots (CRS). Figure 2A and B exhibited the typical and negative CRS findings from Patient 2 and Patient 8, respectively. Nine patients were treated with drugs as detailed in Table 2. Clonazepam, levetiracetam, valproic acid, and oxcarbazepine were used to improve myoclonus and seizures. Buspirone was used to ameliorate ataxia. Among all the drugs, clonazepam was the most frequently used and administered to seven patients. It was followed by baclofen and buspirone, each used in three patients. Physical examination of 10 patients revealed that eight patients exhibited hyperreflexia, while three patients exhibited impaired muscle strength.

**Table 2:** Clinical characteristics of ten ST-1 patients in this study

Patient	P1	P2	P3	P4	P5	P6	P7	P8	P9	P10	
Age/Gender	18/F	13/F	26/F	34/M	44/M	40/F	46/F	40/F	53/F	35/M	
Birthplace	Zhejiang	Jiangsu	Zhejiang	Zhejiang	Fujian	Zhejiang	Zhejiang	Guangdong	Zhejiang	Fujian	
Age at onset	11	7	20	22	28	20	36	25	24	15	
Disease duration	7	6	6	12	16	20	10	15	29	20	
Consanguinity	No	No	No	Yes	Yes	No	No	No	No	Yes	
Initial symptoms	Blurred vision	Blurred vision	Myoclonus, gait ataxia	Gait ataxia, myoclonus	Nystagmus, myoclonus	Paroxysmal bradykinesia	Gait ataxia	Gait ataxia	Gait ataxia	Myoclonus	
Seizures	+	+	-	-	-	-	-	-	-	-	
Myoclonus	+	+	+	+	+	+	+	+	+	+	
Cerebellar ataxia	+	+	Mild	+	+	Mild	+	+	+	+	
Blurred vision	+	+	-	-	-	-	-	+	-	+	
Nystagmus	+	-	-	-	+	-	-	-	+	+	
Hyperreflexia	+	+	+	+	+	+	-	-	+	+	
impaired muscle strength	-	+	+	-	-	-	-	-	-	+	
Other symptoms						Increased eye distance, tongue fibrillation			Restless leg syndrome		
Cherry-red spot	+	+	U	U	-	-	U	-	-	-	
Visual field	U	Defect	U	U	Defect	Defect	U	-	-	-	
OCT	U	Hyperreflex of macular inner layer	U	U	-	-	U	-	-	-	
SARA	U	18	4	18.5	12	3.5	U	U	U	U	
ICARS	U	47	6	52	28	9	U	U	U	U	
MMSE	U	29	30	30	27	26	U	U	U	U	
EEG	U	U	Scattered slow activity	Scattered slow activity	Scattered slow activity	Normal	Normal	U	Normal	U	
EMG	U	U	Abnormal	U	Abnormal	Normal	Abnormal	U	Abnormal	U^a^	
VEP	U	U	U	U	U	U	Prolonged	U	Absence	U	
SSEP	U	U	U	U	U	U	U	U	Giant wave	U	
Brain MRI	Unremarkable	Unremarkable	Unremarkable	Mild cerebellar atrophy and frontotemporal atrophy	Unremarkable	Unremarkable	Frontotemporal atrophy,	Unremarkable	Unremarkable	Unremarkable	
Mutation	c.239 C > T, c.544 A > G	c.544 A > G, c.1021 C > T	Homozygous c.544 A > G	Homozygous c.544 A > G	Homozygous c.544 A > G	c.544 A > G, c.799 C > T	Homozygous c.557 A > G	Homozygous c.544 A > G	Homozygous c.544 A > G	Homozygous c.544 A > G	
Therapy	Clonazepam, levetiracetam	Clonazepam	Clonazepam, baclofen, buspirone	Baclofen, buspirone	Clonazepam, levetiracetam	Oxcarbazepine, clonazepam	Clonazepam, baclofen, buspirone		Valproate sodium	Carbamazepine, clonazepam	

*U* unavailable; *OCT* optical coherence tomography; *SARA* scale for the assessment and rating of ataxia; *ICARS* international cooperative ataxia rating scale; *MMSE* mini-mental state examination; *EEG* electroencephalogram; *EMG* electromyography; *SSEP* somatosensory evoked potential; *VEP* visual evoked potential; *a* the patient underwent tremor electromyography and results showed unremarkable, and his conventional electromyography is unavailable

On ophthalmological examination, defect visual field detects were found in three of the six tested patients. Increased reflectivity of the macular inner layer was only in patients who exhibited cherry-red spots. Six patients had EEG results, three had increased diffuse slow waves in the background, and none had spike waves or polyspike waves (Fig. 2F). Four out of the six patients who underwent EMG assessment had abnormal results. One patient exhibited a slow conduction velocity in the right tibia motor nerve and right sural sensory nerve, one had cervical and lumbar polyradiculopathies, one had sensorimotor peripheral polyneuropathies of both lower extremities, and one had abnormal discharge of motor cortex and lumbar radiculopathy. Patient 7 exhibited prolonged visual evoked potentials in the P100, N75 and N45 components. The somatosensory evoked potential (SEP) of Patient 9 showed a very high amplitude in the P25 and N34 components where the left was 18.1 uV and the right was 51.5 uV, consistent with giant SEP. Her VEP was absent and had poor visual acuity but no CRS. All 10 patients had MRI results and 8 showed unremarkable results (Fig. 2D). Patient 4 showed slight cerebellar atrophy and frontotemporal atrophy, but her MMSE score was 30. Patient 7 showed frontotemporal atrophy but found no intelligence decline. Typical brain MRI and EEG from the patient with the homozygous c.544 A > G genotype were exhibited in Fig. 2.

## Special manifestations

Patient 6 was a 40-year-old female who initiated with paroxysmal bradykinesia at 20 years of age. The patient presented with induced paroxysmal walking difficulty and vague speech, aggravated by nervous and emotion fluctuations. When she was nervous, she manifested body rigidity, difficulty in initiating walking, tremor in the legs and a vague voice. She had no myoclonus, seizures, or visual defects. Initially, she was diagnosed with Parkinson’s disease (PD), and was treated with levodopa, selegiline and amantadine. These drugs did not improve her symptoms. On physical examination, the patient showed no obvious ataxia sign. Her eye movement, limb ataxia and line walking tests were normal. She stammered only if spoke fast. Her SARA and ICARS scores were 3.5 and 9, respectively. Other positive physical examinations included increased eye distance, tongue fibrillation, and hyperreflexia in the lower extremities. Her visual field showed dark spots in both eye fields, but no CRS was found. Blood test, brain MRI, EEG and EMG results of the patient were all unremarkable. Because she presented with PD-like manifestations, DAT-FDG PET was also performed for differential diagnosis. However, DAT-FDG PET result suggested that the distributions of dopamine transporters in the bilateral caudate nucleus and putamen were not obviously abnormal. Her DAT-FDG PET result were shown in Fig. 2H. After diagnosed with ST-1, she was administrated with oxcarbazepine, clonazepam and piracetam, which markedly improved her symptoms of walking, speech, and myoclonus.

### Genotype-phenotype analysis in Taiwan and mainland China

A previous literature review of ST-1 patients showed that the c.544 A > G mutation could be detected only in ST-1 patients in Taiwan and mainland China, and different genetic spectra have been observed in other Asian countries such as Japan, Korean and Indian [9]. Because of the genetic characteristics of Taiwan and mainland China as well as the founder effect of the c.544 A > G mutation, we systemically analyzed the clinical features of ST-1 patients according to their genotypes in Taiwan and mainland China (Table 3). After systematic review, 26 patients from 25 families in mainland China and 26 patients from 20 families in Taiwan were included. In total, 13 different variants were identified in 52 patients. As shown in Table 3, the most frequent genotype was the c.544 A > G homozygote, which was found in 19 out of the 45 families (42.2%). The second most frequent genotype was the c.544 A > G/c.239 C > T compound heterozygote, which was observed in 10 families (22.2%). The genotypes c.544 A > G/c.314_352del, c.544 A > G/c.1021 C > T, c.838 C > T/c.403G > A, c.803 A > G/c.239 C > T and c.544 A > G/ c.956 C > T were each found in two families, each representing 4.4%. The remaining six families had six different genotypes, each comprising 2.2% of the total. The mean AAO of 26 ST-1 patients with homozygous c.544 A > G was 20.0 years (SD 6.0, range 12–33). Ten patients carrying the c.544 A > G/c.239 C > T mutation had a mean AAO of 12.0 years (SD 3.1, range 8–17). We found that ST-1 patients with homozygous c.544 A > G developed the disease at a later age compared to those with c.544 A > G/c.239 C > T (*t* test, *p* < 0.05) or non-homozygous c.544 A > G (*t* test, *p* < 0.05), suggesting the homozygous c.544 A > G would lead to a milder phenotype, and this result was independent of the region (partial correlation, *p* = 0.013). Additionally, CRS occurred in 4.5% of the patients with the homozygous c.544 A > G in the Chinese mainland and Taiwan. This percentage was significantly lower than the CRS occurrence of 70% in patients with the c.544 A > G/c.239 C > T (Fisher’s exact test, *p* < 0.05). No differences of other symptoms were found between the homozygous c.544 A > G and c.544 A > G/c.239 C > T patients (Fisher’s exact test, *p* > 0.05). When comparing patients with homozygous c.544 A > G to those without, CRS occurred in 4.5% and 80.0% respectively (Fisher’s exact test, *p* < 0.05). Furthermore, no correlation between CRS and disease duration was observed (partial correlation, *p* > 0.05).

**Table 3:** Clinical features of ST-1 patients with different genotypes in Taiwan and mainland China

	Homo. c.544 A > G	c.544 A > G; c.239 C > T	c.544 A > G; c.314_352del	c.544 A > G; c.1021 C > T	c.838 C > T; c.403G > A	c.803 A > G; c.239 C > T	c.544 A > G; c.956 C > T	c.544 A > G; c.163 C > T	c.544 A > G; c.619 C > T	c.1118T > C; c.544 A > G	c.544 A > G; c.799 C > T	Homo. c.557 A > G	c.544 A > G; 27.5 kb del
Patients (families)	26(19)	10(10)	2(2)	2(2)	2(2)	2(2)	2(2)	1(1)	1(1)	1(1)	1(1)	1(1)	1(1)
Age, *X* ± SD (N, range)	37.6 ± 7.7 (26, 25–53)	21.5 ± 9.3 (10, 10–36)	16.5 ± 0.7 (2, 16–17)	14.0 ± 1.4 (2, 13–15)	20.0 ± 0 (2, 20–20)	18.0 ± 1.4 (2, 17–19)	34.5 ± 13.4 (2, 25–44)	27.0 (1)	15.0 (1)	19.0 (1)	40.0 (1)	46.0 (1)	11.0 (1)
AAO, *X* ± SD (N, range)	20.0 ± 6.0 (26, 12–33)*	12.0 ± 3.1 (9, 8–17)*	10.5 ± 0.7 (2, 10–11)	8.5 ± 2.1 (2, 7–10)	10.0 ± 0 (2, 10–10)	13.0 ± 1.4 (2, 12–14)	28.0 ± 20.0 (2, 14–42)	12.0 (1)	12.0 (1)	10.0 (1)	20.0 (1)	36.0 (1)	11.0 (1)
SMAV (N)	S(5)M(13) A(5)V(6)	S(0)M(4) A(0)V(3)	S(1)M(0) A(0)V(0)	S(0)M(0) A()V(1)	S(0)M(0) A(1)V(0)	S(2)M(0) A(0)V(0)	S(0)M(1) A(0)V(1)	S(0)M(1) A(0)V(0)	S(1)M(1) A(1)V(1)	S(1)M(0) A(0)V(0)	S(0)M(0) A(0)V(0)	S(0)M(0) A(1)V(0)	S(0)M(0) A(0)V(1)
Seizures	76.9% (20/26)	70% (7/10)	100% (2/2)	100% (2/2)	100% (2/2)	100% (2/2)	100% (2/2)	100% (1/1)	100% (1/1)	100% (1/1)	0% (0/0)	0% (0/0)	0% (0/0)
GTCS	26.7% (4/15)	42.9% (3/7)	50% (1/2)	50% (1/2)	50% (1/2)	50% (1/2)	0% (0/0)	0% (0/0)	100% (1/1)	100% (1/1)	0% (0/0)	0% (0/0)	0% (0/0)
MCS	73.1% (19/26)	70% (7/10)	100% (2/2)	100.0% (2/2)	100% (2/2)	100% (2/2)	50% (1/1)	100% (1/1)	100% (1/1)	100% (1/1)	0% (0/0)	0% (0/0)	0% (0/0)
Myoclonus	96.2% (25/26)	80% (8/10)	100% (2/2)	100% (2/2)	100% (2/2)	100% (2/2)	100% (2/2)	100% (1/1)	100% (1/1)	100% (1/1)	100% (1/1)	0% (0/0)	0% (0/0)
Ataxia	88.5% (23/26)	90% (9/10)	100% (2/2)	100% (2/2)	100% (2/2)	100% (2/2)	100% (2/2)	100% (1/1)	100% (1/1)	100% (1/1)	100% (1/1)	100% (1/1)	100% (1/1)
Impaired vision	72% (18/25)	90% (9/10)	50% (1/2)	100% (2/2)	100% (2/2)	100% (2/2)	100% (2/2)	100% (1/1)	100% (1/1)	100% (1/1)	0% (0/0)	0% (0/0)	100% (1/1)
CRS	4.5% (1/22)*	70% (7/10)*	50% (1/2)	100% (2/2)	100% (2/2)	100% (2/2)	100% (2/2)	100% (1/1)	100% (1/1)	100% (1/1)	0% (0/1)	U	100% (1/1)

*Homo* homozygous; *AAO* age at onset; SMAV: *S* seizures, *M* myoclonus, *A* ataxia, *V* impaired vision; *GTCS* generalized tonic-clonic seizure; *MCS* myoclonus seizures; *CRS* cherry red spot; *U* unavailable

*Fisher’s exact test, *p* < 0.05

## Discussion

In the current study, we documented the genetic and clinical characteristics of 10 ST-1 patients. A high frequency of the homologous c.544 A > G genotype was reported, which was caused by the founder effect of the c.544 A > G mutation. We also confirmed that macular CRS was rare among homologous c.544 A > G ST-1 patients, which occurred much less often in heterozygous c.544 A > G patients or patients harboring other mutations in Taiwan and mainland China. Although seven prior studies in China have reported on ST-1 patients, most of them were case studies. Only two studies enrolled four and five ST-1 patients from mainland China respectively [8, 9]. Therefore, our study was the largest investigation of ST-1 in mainland China.

A high percentage of the homozygous c.544 A > G variant was found in Taiwan, with a reported percentage of 83.3% [13]. Intriguingly, except for in Taiwan and mainland China, the c.544 A > G mutation was rarely found in other populations [9], which suggested that the ST-1 patients in Taiwan and southeast China shared the similar genetic background. Among our 6 ST-1 patients harboring homozygous c.544 A > G, AAO was 22.3 years, and it was 19.3 in 15 patients with homozygous c.544 A > G in Taiwan [13], indicating a similar onset. Notably, CRS was observed in 28.5% of the tested patients in the current study, none was detected in the patients with the homozygous c.544 A > G mutation, whereas the incidence was 17.6% in Taiwan patients [13]. Vision impairment was detected in 50% of our ST-1 patients and 82.3% of Taiwan patients [13]. Even within Asia, visual impairment exhibited a variety across the populations. In India, no ST-1 patients presented with visual impairment [14]. However, in Japan, half of the ST-1 patients manifested visual impairment [15, 16]. Among all the patients, Patient 6 exhibited a distinguished phenotype characterized by parkinsonism. Initially, she presented with paroxysmal bradykinesia and was misdiagnosed with Parkinson’s disease at her first visit. However, further examination showed that she also had myoclonus, mild cerebellar ataxia, and hyperreflexia, which could not be explained by Parkinson’s disease. In addition, her DAT-FDG PET scan was normal. Although she exhibited no CRS, ST-1 should be considered, and WES was necessary.

The combined analysis of 52 patients from Taiwan and mainland China confirmed that later AAO and lower CRS occurrence were significantly different in ST-1 patients with the homozygous c.544 A > G mutation. This finding was consistent with the results of the previous study [13]. However, when comparing AAO and CRS among different populations, the difference became too small to be detected statistically. It can be considered that genetic mutations, rather than population background, play a more important role in sialidosis. Additionally, CRS incidence was more profoundly influenced by genetic mutations rather than by disease duration. Prior research suggested that the c.544 A > G mutation occurred outside the catalytic region of the enzyme, resulting in no apparent structural change, and nearly half of the residual activity remaining [5]. However, due to the limited number of reported ST-1 patients, contradictory conclusions might arise when the sample size was enlarged.

Notably, abnormal EEG results were not observed in the current study, despite one of our patients having a confirmed history of GTCS. Fan et al. reported that Asian ST-1 patients had a lower percentage of abnormal EEGs than non-Asian patients did [17]. To some extent, these results were consistent with our findings. Since visual impairment was not evident in the current cohort, VEP and SEP were not obtained for all the patients, representing limitations of our study. Previously, Lai suggested that VEP could provide a more sensitive assessment when ST-1 patients present atypical manifestations [13]. The giant SEP has been considered an objective assessment correlated with PME and constituted 100% of sialidosis patients [17–19]. However, giant SEPs were not specific enough to discriminate sialidosis from other diseases with PME. Two out of 10 ST-1 patients in our cohort exhibited abnormal brain MRI findings. Both patients showed frontotemporal atrophy, but neither developed obvious cognitive decline. Frontotemporal atrophies were also discovered in the previous report [13]. Diffuse brain atrophy could also be observed in ST-1 patients, but intelligent abnormities were rarely reported [15, 20, 21]. Interestingly, although ataxia could be detected in all patients in the current cohort, only one patient had slight cerebellar atrophy.

Currently, there is still no specific treatment for sialidosis. Despite being an enzyme-defective disease, enzyme replacement therapy has failed in animal experiments due to the severe immune response [22]. Two previous reports demonstrated that perampanel might alleviate myoclonus severity [23, 24]. Moreover, a recent report of deep brain stimulation (DBS) showed that ST-1 patients with intractable myoclonus benefited from it [25].

## Conclusion

Our study contributed to gaps in the clinical and genetic features of ST-1 patients in southeastern mainland China and we hope the results could provide a deeper understanding of this disease.

## Data Availability

The datasets generated and analyzed in this study are not publicly available to assure participant privacy. The datasets can only be provided upon reasonable request to the corresponding author.

## References

[1] Cantz M, Gehler J, Spranger J. Mucolipidosis I: increased sialic acid content and deficiency of an alpha-N-acetylneuraminidase in cultured fibroblasts. Biochem Biophys Res Commun. 1977;74:732. 836321 10.1016/0006-291x(77)90363-1

[2] Spranger J, Cantz M, Mucolipidosis I. The cherry red-spot–myoclonus syndrome and neuraminidase deficiency. Birth Defects Orig Artic Ser. 1978;14:105. 728556

[3] Lowden JA, O’Brien JS. Sialidosis: a review of human neuraminidase deficiency. Am J Hum Genet. 1979;31:1. 107795 PMC1685665

[4] Bonten ErikJ. Novel mutations in lysosomal neuraminidase identify functional domains and determine clinical severity in sialidosis. Hum Mol Genet. 2000;9:2715. 11063730 10.1093/hmg/9.18.2715

[5] Lukong KE. Characterization of the sialidase molecular defects in sialidosis patients suggests the structural organization of the lysosomal multienzyme complex. Hum Mol Genet. 2000;9:1075. 10767332 10.1093/hmg/9.7.1075

[6] Chin SJ, Fuller M. Prevalence of lysosomal storage disorders in Australia from 2009 to 2020. Lancet Reg Health - West Pac. 2022;19:100344. 35024668 10.1016/j.lanwpc.2021.100344 PMC8671750

[7] Caciotti A, Melani F, Tonin R, Cellai L, Catarzi S, Procopio E, et al. Type I sialidosis, a normosomatic lysosomal disease, in the differential diagnosis of late-onset ataxia and myoclonus: an overview. Mol Genet Metab. 2020;129:47. 31711734 10.1016/j.ymgme.2019.09.005

[8] Han X, Wu S, Wang M, Li H, Huang Y, Sui R. Genetic and clinical characterization of mainland Chinese patients with sialidosis type 1. Mol Genet Genomic Med. 2020;8:e1316. 32453490 10.1002/mgg3.1316 PMC7434748

[9] Lv R, Li T, Zhang Y, Shao X, Wang Q, Jin L. Clinical and genetic characteristics of type I sialidosis patients in mainland China. Ann Clin Transl Neurol. 2020;7:911. 32472645 10.1002/acn3.51058 PMC7318099

[10] Du YC, Dong Y, Cheng HL, Li QF, Yang L, Shao YR, et al. Genotype-phenotype correlation in 667 Chinese families with spinocerebellar ataxia type 3. Parkinsonism Relat Disord. 2020;78:116. 32814229 10.1016/j.parkreldis.2020.07.024

[11] Cheng HL, Shao YR, Dong Y, Dong HL, Yang L, Ma Y, et al. Genetic spectrum and clinical features in a cohort of Chinese patients with autosomal recessive cerebellar ataxias. Transl Neurodegener. 2021;10:40. 34663476 10.1186/s40035-021-00264-z PMC8522248

[12] Wang F, Lin L, Hu J, Zhang J, Wang K. Neurophysiolgical implications in sialidosis type 1: a case report. Int J Neurosci. 2022;132:589. 32988250 10.1080/00207454.2020.1829615

[13] Lai SC, Chen RS, Wu Chou YH, Chang HC, Kao LY, Huang YZ, et al. A longitudinal study of Taiwanese sialidosis type 1: an insight into the concept of cherry-red spot myoclonus syndrome. Eur J Neurol. 2009;16:912. 19473359 10.1111/j.1468-1331.2009.02622.x

[14] Neeraja K, Holla VV, Prasad S, Surisetti BK, Rakesh K, Kamble N, et al. Sialidosis type I without a Cherry Red Spot- Is there a genetic basis? J Mov Disorders. 2021;14:65. 10.14802/jmd.20083 PMC7840231 33121223

[15] Sekijima Y, Nakamura K, Kishida D, Narita A, Adachi K, Ohno K, et al. Clinical and serial MRI findings of a sialidosis type I patient with a novel missense mutation in the NEU1 gene. Intern Med. 2013;52:119. 23291686 10.2169/internalmedicine.52.8901

[16] Uchihara T, Ohashi K, ichi, Kitagawa M, Kurata M, Nakamura A, Hirokawa K, et al. Sialidosis type I carrying V217M/G243R mutations in lysosomal sialidase: an autopsy study demonstrating terminal sialic acid in lysosomal lamellar inclusions and cerebellar dysplasia. Acta Neuropathol. 2010;119:135. 19415310 10.1007/s00401-009-0544-x

[17] Fan SP, Lee NC, Lin CH. Clinical and electrophysiological characteristics of a type 1 sialidosis patient with a novel deletion mutation in NEU1 gene. J Formos Med Assoc Taiwan Yi Zhi. 2020;119:406. 31371146 10.1016/j.jfma.2019.07.017

[18] Lu CS, Ng SH, Lai SC, Kao LY, Liu L, Lin WY, et al. Cortical damage in the posterior visual pathway in patients with sialidosis type 1. Brain Imaging Behav. 2017;11:214. 26843009 10.1007/s11682-016-9517-6

[19] Huang YZ, Lai SC, Lu CS, Weng YH, Chuang WL, Chen RS. Abnormal cortical excitability with preserved brainstem and spinal reflexes in sialidosis type I. Clin Neurophysiol. 2008;119:1042. 18343720 10.1016/j.clinph.2008.01.023

[20] Kersten HM, Roxburgh RH, Danesh-Meyer HV, Hutchinson DO. Optical coherence tomography findings in a patient with type 1 sialidosis. J Clin Neurosci. 2016;31:199. 27052257 10.1016/j.jocn.2016.02.015

[21] Gultekin M, Bayramov R, Karaca C, Acer N. Sialidosis type I presenting with a novel mutation and advanced neuroimaging features. Neurosciences. 2018;23:57. 29455223 10.17712/nsj.2018.1.20170328 PMC6751914

[22] Wang D, Bonten EJ, Yogalingam G, Mann L, d’Azzo A. Short-term, high dose enzyme replacement therapy in sialidosis mice. Mol Genet Metab. 2005;85:181. 15979029 10.1016/j.ymgme.2005.03.007

[23] Hu SC, Hung KL, Chen HJ, Lee WT. Seizure remission and improvement of neurological function in sialidosis with perampanel therapy. Epilepsy Behav Case Rep. 2018;10:32. 29977792 10.1016/j.ebcr.2018.02.005 PMC6030028

[24] So ECT, Mak CM, Ng GSF, Tsui KW, Ma KH, Yeung WL. Reduction in myoclonus and ataxia following the use of perampanel in patient with sialidosis type 1. Pediatr Neurol. 2020;109:91. 32299749 10.1016/j.pediatrneurol.2020.03.004

[25] Liu J, Ouyang Y, Lv H, et al. Deep brain stimulation for myoclonus in sialidosis I. Parkinsonism Relat Disord. 2023;111:105434. 37167833 10.1016/j.parkreldis.2023.105434

